# Implementation of Highly Reliable Contacts for RF MEMS Switches

**DOI:** 10.3390/mi15010155

**Published:** 2024-01-20

**Authors:** Lili Jiang, Lifeng Wang, Xiaodong Huang, Zhen Huang, Min Huang

**Affiliations:** 1 Key Laboratory of MEMS of the Ministry of Education, School of Electronic Science and Engineering, Southeast University, Nanjing 210096, China; 230199026@seu.edu.cn; 2 Nanjing Electronic Devices Institute, Nanjing 210016, China; hertzen_mems@126.com (Z.H.); sensorsss@163.com (M.H.)

**Keywords:** RF MEMS switch, highly reliable contacts, shape of contacts, lifetime

## Abstract

A contact is the key structure of RF MEMS (Radio Frequency Microelectromechanical System) switches, which has a direct impact on the switch’s electrical and mechanical properties. In this paper, the implementation of highly reliable contacts for direct-contact RF MEMS switches is provided. As a soft metal material, gold has the advantages of low contact resistance, high chemical stability, and mature process preparation, so it is chosen as the metal material for the beam structure as well as the contacts of the switch. However, a Pt film is used in the bottom contact area to enhance the reliability of the contact. Three kinds of contacts with various shapes are fabricated using different processes. Particularly, a circular-shaped contact is obtained by dry/wet combined processes. The detailed fabrication process of the contacts as well as the Pt film on the bottom contact area are given. The experimental test shows that the contact shape has little effect on the RF performance of the switches. However, the circular contact shows better reliability than other contacts and can work well even after 1.2 × 10^9^ cycles.

## 1. Introduction

RF MEMS is a radio frequency device made using MEMS technology. Compared with traditional RF devices, RF MEMS devices inherit many advantages of MEMS devices, including their small size, insensitivity to acceleration, and low DC power consumption. In addition, RF MEMS devices can also be integrated with traditional silicon-based and gallium arsenide-based circuits, enabling the miniaturization of RF processing systems [1]. An RF MEMS switch is a microcomponent that is used to control the on/off of RF signals. It contains both an RF structure and a mechanical structure, which has the advantages of excellent linearity, very low power consumption, and pretty good insertion loss and isolation [2,3]. RF MEMS switches can be and are already widely used in RF or microwave applications, such as tunable antennas, phase shifters, phased array radars, multi-band RF communications, and automatic countermeasure systems [4].

RF MEMS switches contain both an RF structure and a mechanical structure. According to the different connection methods of movable beams, RF MEMS switches can be divided into parallel and series types. Then, according to the different isolation methods of RF signals, RF MEMS switches can further be divided into direct contact types and capacitive coupling types. Therefore, in terms of RF structure, RF MEMS switches can be divided into four basic types: series contact, series capacitive, shunt contact, and shunt capacitive. From the perspective of mechanical structure, the driving methods for RF MEMS switches include electrostatic drive, thermal drive, electromagnetic drive, and piezoelectric drive [5,6,7,8]. Due to the advantages of having a simple driving structure and fast response speed, electrostatic driving has become the preferred driving method for RF MEMS switches [1]. The reported electrostatic drive switches include all four types: series contact, series capacitor, shunt contact, and shunt capacitive [9,10,11,12,13]. Among them, electrostatic-driven direct contact switches have exhibited several advantages over other types of switches, such as better isolation in the commonly used low-frequency band and higher reliability [14].

The contact structure is the key structure of electrostatic-driven direct-contact RF MEMS switches, which not only affects the RF performance of the switch but is also closely related to the long-term reliability of the switch. In order to achieve better performance and reliability, various contact materials have been studied and compared. In addition to conventional metal materials, researchers have also attempted to use alloys, metal oxides, and nanomaterials [15,16,17,18,19]. An Al–Sc alloy is used to replace the traditional Au–Au contact, which can greatly improve the hardness of the contact and, thus, improve the reliability of the switch [15]. The implementation of RuO_2_–Au contact metallurgy into an existing design showed an improved switch lifetime of over 10 billion cycles [16]. Sawant V et al. evaluated and selected contact materials capable of achieving low contact resistance with high-reliability switches. The analytical and experimental results show that graphene, gold, and rhodium emerge as the top-ranked contact materials [17]. Au–Au/CNT contacts have been proposed to improve the reliability and high-power handling capability of ohmic contact-type RF-MEMS switches [18]. A review of microcontact physics, materials, and failure mechanisms in direct-contact RF MEMS switches is provided by Basu A et al. [19].

In addition to contact materials, different contact structures have also been designed to improve the reliability and performance of the switches. By designing a ball grid array dimple in the contact structure, the switch lifetime is greatly extended [20]. Another design of multi-point contact in the contact structure helps to improve the power handling of the switches [21]. The relationship between the contact area and the RF power handling of the switch has been studied by Anuroop et al. [22]. Different from conventional vertical-moving contact structures, laterally moving contact structures based on a monocrystalline silicon material also exhibit high reliability and high-power handling [23,24].

In this paper, firstly, an electrostatically driven direct-contact RF MEMS switch is designed. Then, a highly reliable contact material as well as its process implementation method are provided. On this basis, three different contact shapes are designed and fabricated using three different process methods. Finally, the RF and mechanical properties of different contact morphology switches are measured and compared. Among them, the circular contact formed using the dry/wet mixed process shows the longest lifespan.

## 2. Switch Design

**Figure 1:**
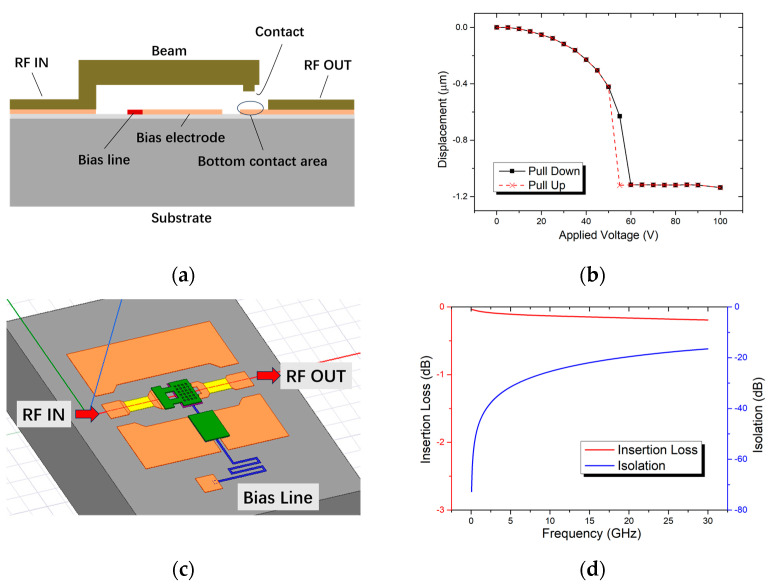
RF MEMS switch design. (**a**) Schematic view of the switch; (**b**) simulated actuation voltage of the designed switch; (**c**) simulation model of the switch in HFSS (High-Frequency Simulation Software 15.0); (**d**) simulated RF performances of the designed switch.

Here, the RF MEMS switch structure adopts the most common electrostatic-driven series contact switch. As shown in Figure 1a, the anchor area of the cantilever beam is connected with the RF signal input end. The bottom electrode is directly below the cantilever beam. A voltage is applied between the cantilever beam and the bottom electrode to generate electrostatic force to control the deflection of the cantilever beam. There are two contacts at the end of the cantilever beam. When the cantilever beam is pulled down, the contacts are connected between the input and output ends of the RF signal.

The RF MEMS switch contains both an RF structure and a mechanical structure. Therefore, the design of the RF MEMS switch can be divided into two parts: one is the mechanical performance design, and the other is the RF performance design. Both the transmission line and the switch structure are made of gold. The elastic modulus of gold is 78 GPa, and the Poisson’s ratio is 0.44 [25]. The thickness of the transmission line is 1μm, the thickness of the cantilever structure is 5 μm, and the size of the upper electrode plate of the cantilever beam is 100 × 80 μm. The mechanical performance of the designed switch is simulated in CoventorWare. The simulated driving voltage is 60 V, as shown in Figure 1b, and the switching time is 12 μs. In terms of RF design, the switch uses an HRS (high-resistance silicon) wafer as its substrate with a thickness of 400 μm. The resistivity of the HRS substrate is greater than 6000 Ω·cm. Figure 1c shows the simulation model of the switch in HFSS, and Figure 1d gives the simulated insertion loss and isolation. The simulated insertion loss of the switch is 0.164 db@20 GHz, and the isolation is 20.058 db@20 GHz. Here, the contact resistances between the contacts and the transmission line are not considered in the insertion loss simulation. Some key parameters of the switch are listed in Table 1.

## 3. Implementation of Highly Reliable Contacts

### 3.1. Material Selection for Conctact Structure

The contact structure is one of the key parts of the contact-type RF MEMS switch, which directly affects the performance and reliability of the device. The contact structure of the switch includes the contacts on the movable beam and the bottom contact area. In RF MEMS switches, the selection of contact materials mainly considers or evaluates the parametric characteristics of the material, such as electrical resistivity, hardness, melting point, and chemical stability. Among them, resistivity determines the RF performance of the switch, while hardness, melting point, and chemical stability are related to the mechanical properties and reliability of the switch.

As a soft metal material, gold has the advantages of low electrical resistivity, low contact resistance, high thermal conductivity, a high melting point, mature process preparation, and strong oxidation resistance. In RF MEMS switches, gold has become the primary choice for beam structures. Although gold materials have excellent physical and chemical properties, the design of the gold-to-gold contact structure does not benefit the lifetime of switches. This is because when two soft materials come into contact, the adhesion in the contact area is quite high, which makes the switch prone to contact wear, contact material transfer, and permanent contact adhesion failure.

Pt series metals, as hard metal materials, have the advantages of high softening temperature, high corrosion resistance, and high wear resistance [26]. If the contacts and the bottom contact area are all made of Pt series metals, the plastic deformation of the contacts can be decreased, and the power processing and reliability of the switch can be significantly improved. However, this design will cause problems such as increased contact resistance, increased driving voltage, and increased fabrication difficulty.

After considering factors such as contact resistance, long-term reliability, and fabrication implementation, we propose a solution for the contact material, which is to use gold for the contacts and Pt series hard metal for the bottom contact area.

### 3.2. Fabrication Process of Bottom Contact Area

**Figure 2:**
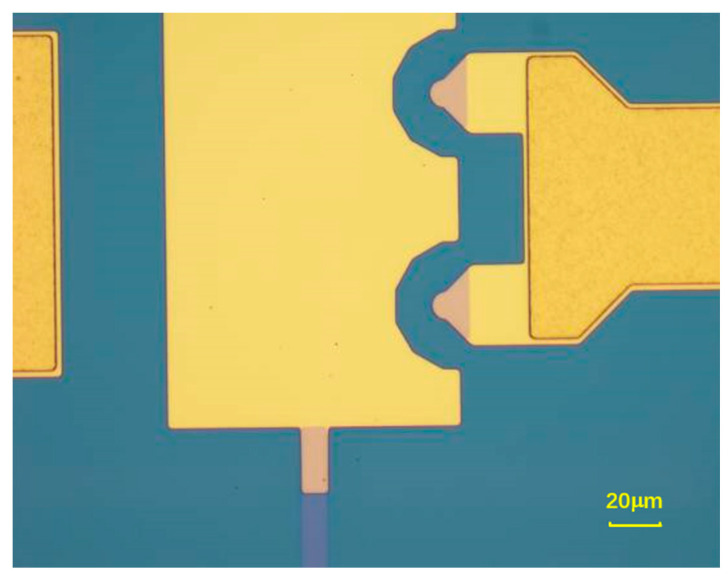
Bottom contact area covered with Pt film.

Here, a Pt layer is deposited in the bottom contact area to enhance the reliability of the contact structure. Pt metals belong to hard metals, and the stress of Pt films grown by a micromachining process is relatively high. Therefore, only the actual contact places are covered with Pt metal, which can avoid stress caused by large areas of the hard metal. The process flow of the Pt bottom contact area is as follows: First, a 200 nm Pt layer is deposited by a sputtering process with a deposit rate of 8 Å/s, and then the pattern of the Pt contact area is photolithographed. Finally, the sputtered Pt layer is etched using ion beam etching. The completed Pt contact area is shown in Figure 2.

During the Pt film growth process, methods like increasing the substrate temperature and negative voltage-biased sputtering are used to improve the film quality as well as enhance the adhesion between the Pt layer and the underlying layer. Firstly, increasing the substrate temperature is beneficial for improving the adhesion between the Pt layer and the underlying layer. As the substrate temperature increases, the diffusion between the Pt particles and base atoms becomes stronger, resulting in tighter bonding between the Pt layer and the base layer. Secondly, negative voltage-biased sputtering is also beneficial for improving adhesion and film quality. Negatively biased voltage can form an accelerating electric field near the substrate. So, the ionized Pt particles will be accelerated to higher energy, which helps to enhance the adhesion and quality of sputtered Pt films.

### 3.3. Shapes and Fabrication Processes of Contacts

The contacts are located below the end of the cantilever beam. By sputtering and electroplating on the surface of the patterned sacrificial layer, the cantilever beam and its contacts are formed at the same time. Both wet etching and dry etching can be used to make the contacts of the switch, but the contact morphology is different. Here, we use dry etching, wet etching, and dry/wet mixed processes to fabricate the switch contacts, and three different types of contacts are obtained.

#### 3.3.1. Type A: Flat Contacts

**Figure 3:**
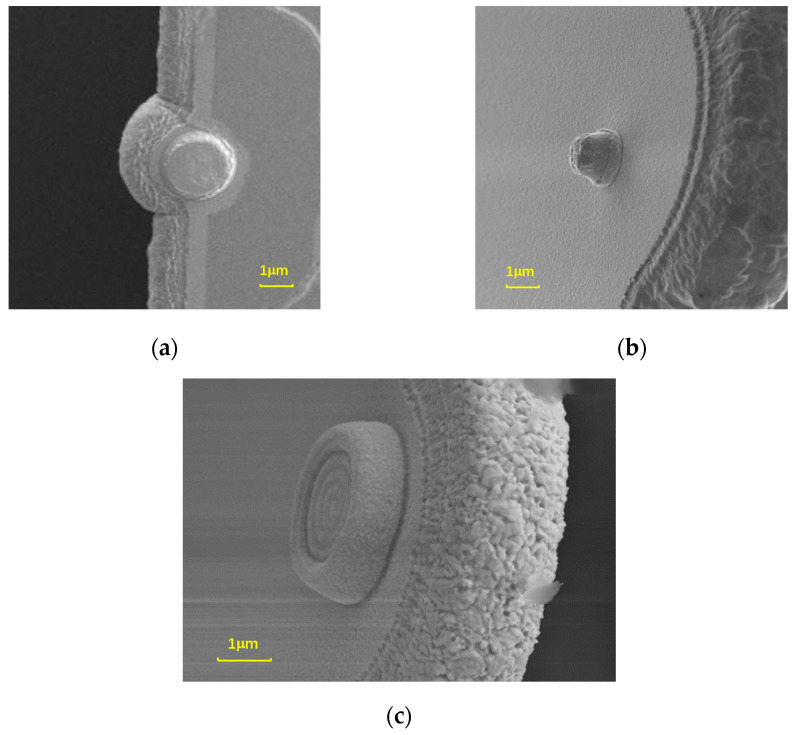
SEM (scanning electron microscope) images of three contacts with different shapes: (**a**) flat contact; (**b**) bump-type contact; (**c**) circular contact.

Flat contacts are formed using the dry etching process. The sacrificial layer is PECVD-deposited 1.5 μm thick SiO_2_ with a deposit rate of 72 Å/s. Then, a 4 μm thick photoresist layer is coated on the sacrificial layer and exposed to an exposure dose of 800 mJ. Next, CHF_3_ gas and oxygen are used to etch the SiO_2_ layer in a dry way to form the contact pits. It is easy to control the etching rate of dry etching by setting its process parameter; hence, the bottom surface of the etched area can be uniform and flat. Here, the etching power is set to 100 W, and the cavity pressure is set to 10 mTor. The dry etching rate of the SiO_2_ layer is obtained at 221 Å/min. Using the dry etching process, shallow, flat contacts are obtained. Here, the contact pits are etched to 0.5 μm in depth. The fabricated flat contact is shown in Figure 3a.

#### 3.3.2. Type B: Bump-Type Contacts

Bump-type contacts are formed using the wet etching process. The sacrificial layer is PSG (phospho-silicate glass) with a thickness of 1.5 μm. BOE (buffered oxide etch, 30% NH_4_F, 8% HF, 62% H_2_O) wet etching is used to etch the sacrificial layer to form the contact pit, of which the depth is about 1 μm. Figure 3b shows the fabricated bump-type contact. As can be seen, the surface roughness of the bump-type contact is relatively larger than that of the flat contact. In other words, the surface etched by BOE is rougher than that of dry etching.

#### 3.3.3. Type C: Circular Contacts

In order to improve the contact effect of the contacts and the long-term reliability of the switch, a circular-type contact is designed here. The circular contact has a larger effective contact area compared to flat and bump-type contacts. In other words, the switch with circular contacts will have a smaller contact pressure and contact current density. Moreover, the middle part of the circular contact is lower than the surrounding part, so the middle part can become a substitute after the circular contact area is worn, which can effectively improve the lifetime of the contact.

Firstly, a SiO_2_ sacrificial layer is deposited. Then, a pit with a depth of 0.5 μm is etched on the surface of the sacrificial layer by dry etching. Next, inside the pit, a circular region is photolithographed, and the depth of the circular region is pushed down 0.3 μm by wet etching. After the photoresist on the surface is removed, it is put into a corrosive solution to smooth the sharp edges and corners inside the circular contact structure. The total thickness of the circular contact is controlled to be 1μm. The fabricated circular contact can be seen in Figure 3c.

### 3.4. Fabrication Processes for the Switch

**Figure 4:**
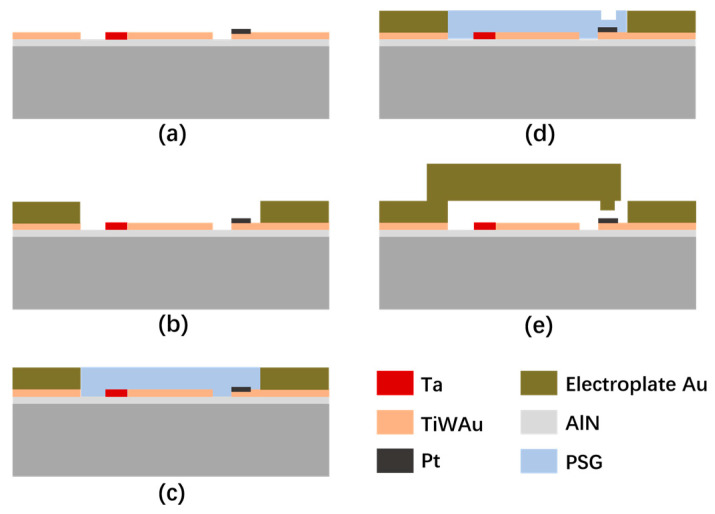
Fabrication process flow of the switch. (**a**) Bottom electrode, DC-biased line, and Pt contact layer are deposited and patterned; (**b**) transmission line is formed; (**c**) sacrificial layer is deposited and patterned; (**d**) sacrificial layer is partly etched; (**e**) anchor, cantilever beam, and contact are formed at the same time, and the switch is released.

**Figure 5:**
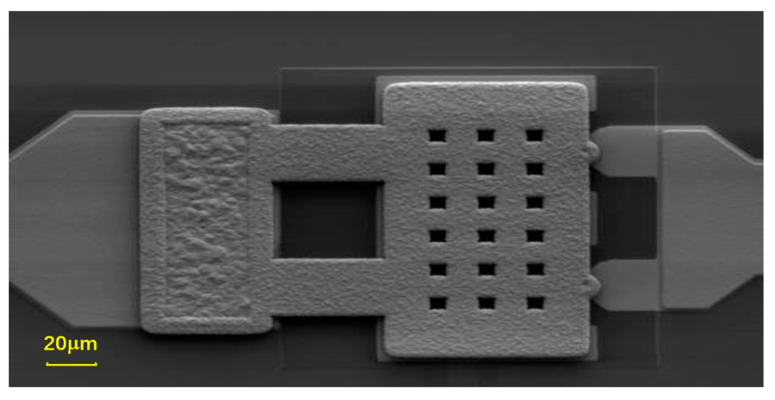
SEM image of the fabricated switch.

Figure 4 depicts the fabrication process flow of the switch. Here, a typical surface sacrificial layer process is used to fabricate the switch. Firstly, the bottom electrode is formed by sputtering 1 μm TiWAu, and the DC bias line is formed by sputtering 1 μm Ta, and then the bottom contact area Pt layer is sputtered and patterned, as shown in Figure 4a. Secondly, the transmission line is formed by electroplating 1 μm Au, as depicted in Figure 4b. Thirdly, Figure 4c shows that the sacrificial layer is deposited on the surface and patterned. Fourthly, Figure 4d shows that the sacrificial layer is partly etched by the dry and/or wet etching process, as described in Section 3.3. Next, the anchor, the cantilever beam, and the contact are formed at the same time by electroplating 5 μm Au, and, finally, the PSG layer is removed and the cantilever beam is released, as depicted in Figure 4e. The SEM scanning diagram of the fabricated switch is shown in Figure 5, and the contacts can be realized by the three different process methods mentioned above.

## 4. Measurement Results and Comparisons

### 4.1. RF Performance Comparison

**Figure 6:**
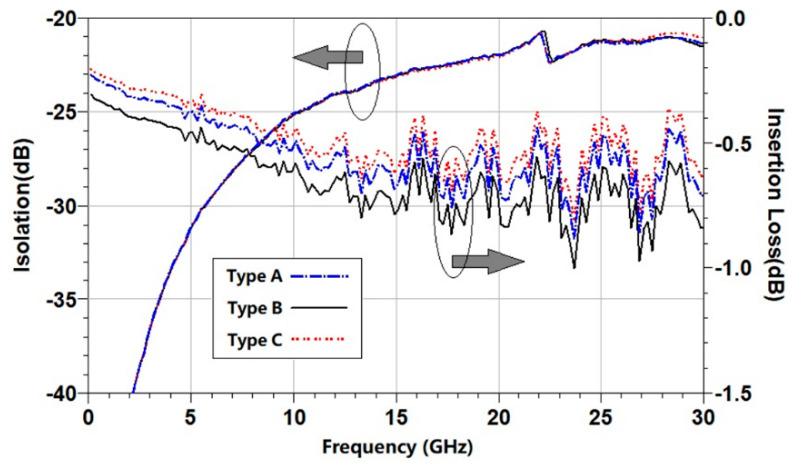
RF testing results of the switches with three different contacts.

The testing platform for RF performance includes a vector network analyzer (Agilent N5242A, Agilent Technologies, Inc., Santa Clara, CA, USA) and an RF probing station. Under the same actuation voltage, the RF performance of the switches with different contacts is tested. The measured results are shown in Figure 6. The insertion loss of type A (flat contact) is −0.72@30 GHz, and the isolation is −21.1@30 GHz; the insertion loss of type B (bump-type contact) is −0.84@30 GHz, and the isolation is −21.1@30 GHz; the insertion loss of type C (circular contact) is −0.63@30 GHz, and the isolation is −20.8@30 GHz. The test results show that the isolations of the three switches are nearly the same, while the insertion losses are slightly different. As can be seen, type C exhibits the smallest insertion loss, type B has the largest insertion loss, and type A is between them. Because they have exactly the same structures, the insertion losses are mainly related to their contact resistances.

In order to compare the contact resistances, the same driving voltage is applied to these switches for the three different types of contacts. Under the same driving voltage, the electrostatic force received by the switch beam is the same, but different switch contacts have different contact resistance due to different contact surfaces and different pressure per unit area. The measured contact resistances of the RF MEMS switches with type A contact, type B contact, and type C contact are 1.3 Ω, 1.8 Ω, and 1.1 Ω, respectively. After removing the 0.2 Ω resistance of the transmission line, the contact resistances of the three types of contacts are calculated as 1.1 Ω, 1.6 Ω, and 0.9 Ω, respectively. The measured resistances are consistent with the insertion loss results.

### 4.2. Lifetime Comparison

**Figure 7:**
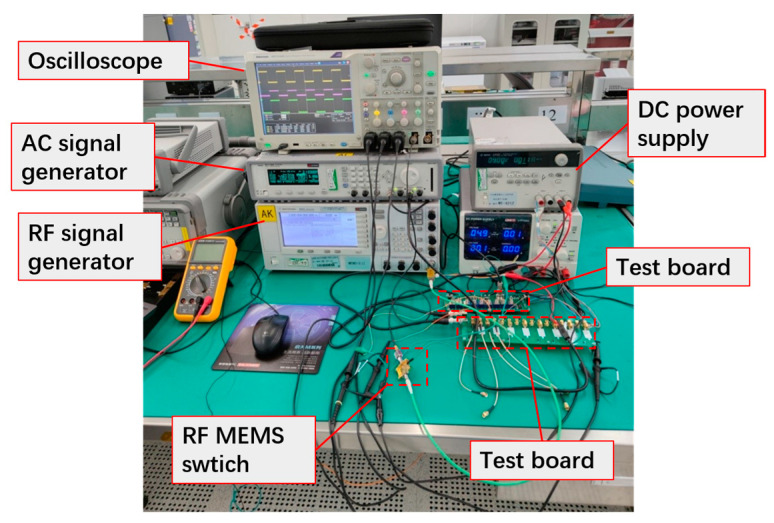
Lifetime test platform for the three types of switches.

Figure 7 shows the test platform for the mechanical performance of the switches. The entire testing platform is in a room-temperature environment. The function generator and the linear amplifier generate square wave signals with adjustable amplitude and frequency for driving the RF MEMS switches. The RF signal generator sends a 2.4 GHz RF signal to the input port of the switch. The RF signal at the output port of the switch is converted into a DC signal by an RF detector. The square wave driving signal and the DC signal from the RF detector are simultaneously sent to the oscilloscope.

Here, the square wave driving voltage used to test the reliability of the switches is set to 60 V and 1 kHz. The switch with type A contact has an unstable switching output signal after 1.9 × 10^7^ times, indicating that it has an unstable contact state and failure after working for a certain number of times. The switch with a type B contact works stably before 3.5 × 10^8^ times and then fails, resulting in a short-circuit failure. In comparison, the switch with a type C contact still works stably after 14 days of continuous tests, which means the measured lifetime of the type C switch is at least 1.2 × 10^9^.

**Figure 8:**
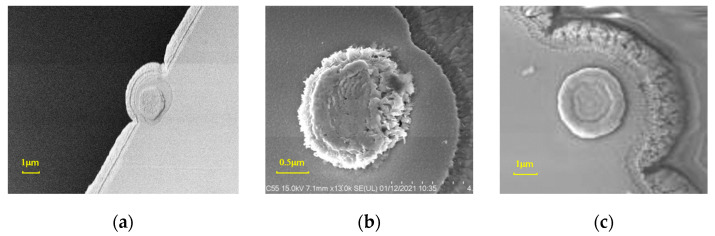
Morphology of the switch contacts after the reliability test: (**a**) flat contact; (**b**) bump-type contact; (**c**) circular contact.

After the lifetime test, the switches are dissected for further analysis. An anatomical analysis reveals that the morphologies of the switch contacts have changed after the reliability test. As shown in Figure 8, type A contact is squeezed flatter, resulting in poor contact failure under a certain voltage. The type B contact is severely deformed after the repeated contacts, and the contact is partially adhered to the bottom contact area, resulting in the short-circuit failure of the switch. The type C contact did not fail after 1.2 × 10^9^ times of operation, and the circular part was partly deformed because of too many contact times. The reliability comparison test indicates that the circular contact can effectively improve the lifetime of the RF MEMS switch.

A comparison of RF performance and lifetime between this work and previously reported direct contact switches is listed in Table 2. It can be seen that the switches proposed in this article have good RF performance over a wide operating frequency. In terms of reliability, type C showed a higher lifetime than type A and type B. At the same time, type C also exhibits top-level reliability when compared to previously reported switches.

## 5. Conclusions

In this paper, the implementation of highly reliable contacts for direct-contact RF MEMS switches is provided. As gold has the advantages of low contact resistance, high chemical stability, and mature process preparation, it is chosen as the metal material for the beam structure as well as the contacts of the switch. However, a Pt film is used in the bottom contact area to enhance the reliability of the contact. By using dry etching, wet etching, and dry/wet mixed processes, three kinds of switch contacts with different shapes, namely, flat contact, bump-type contact, and circular contact, are obtained. The detailed fabrication process of the contacts as well as the Pt film on the bottom contact area are given. A comparative test of the RF performance of RF MEMS switches with three kinds of contacts shows that the contact morphology has little effect on the contact resistance and RF performance of the switch. The long-term reliability test results under the same driving voltage show that the circular contact has better reliability performance among the three contact structures, and a lifetime of at least 1.2 × 10^9^ cycles is obtained.

## Figures and Tables

**Table 1:** Design parameters of the switch.

Parameters	Value
Beam thickness	5 μm
Beam spring size	40 × 20 μm
Beam electrode size	80 × 100 μm
Electrostatic gap	1.5 μm
Gap/Signal/Gap	40 μm/80 μm/40 μm
Actuation voltage	60 V
Chip size	1000 μm × 1200 μm

**Table 2:** Comparison of RF performance and lifetime between this work and previously reported ones.

Reference		Insertion Loss (dB)	Isolation (dB)	Actuation Voltage (V)	Lifetime
[15]		−0.9@6 GHz	−24@6 GHz	75	NULL
[16]		−0.4@10 GHz	−28@10 GHz	120	10 billion
[18]		−0.7@40 GHz	−18@40 GHz	90	9100
[27]		NULL	−40@8 GHz	25	500 million
This work	Type A	−0.72@30 GHz	−21.1@30 GHz	60	19 million
	Type B	−0.84@30 GHz	−21.1@30 GHz		350 million
	Tpye C	−0.63@30 GHz	−20.8@30 GHz		>1.2 billion

## Data Availability

Data are contained within the article.
